# PP2A and cancer epigenetics: a therapeutic opportunity waiting to happen

**DOI:** 10.1093/narcan/zcac002

**Published:** 2022-02-01

**Authors:** Samantha L Tinsley, Brittany L Allen-Petersen

**Affiliations:** Department of Biological Sciences, Purdue University, West Lafayette, IN 47907, USA; Purdue University Interdisciplinary Life Sciences, Purdue University, West Lafayette, IN 47907, USA; Department of Biological Sciences, Purdue University, West Lafayette, IN 47907, USA; Purdue University Center for Cancer Research, Purdue University, West Lafayette, IN 47907, USA

## Abstract

The epigenetic state of chromatin is altered by regulators which influence gene expression in response to environmental stimuli. While several post-translational modifications contribute to chromatin accessibility and transcriptional programs, our understanding of the role that specific phosphorylation sites play is limited. In cancer, kinases and phosphatases are commonly deregulated resulting in increased oncogenic signaling and loss of epigenetic regulation. Aberrant epigenetic states are known to promote cellular plasticity and the development of therapeutic resistance in many cancer types, highlighting the importance of these mechanisms to cancer cell phenotypes. Protein Phosphatase 2A (PP2A) is a heterotrimeric holoenzyme that targets a diverse array of cellular proteins. The composition of the PP2A complex influences its cellular targets and activity. For this reason, PP2A can be tumor suppressive or oncogenic depending on cellular context. Understanding the nuances of PP2A regulation and its effect on epigenetic alterations can lead to new therapeutic avenues that afford more specificity and contribute to the growth of personalized medicine in the oncology field. In this review, we summarize the known PP2A-regulated substrates and potential phosphorylation sites that contribute to cancer cell epigenetics and possible strategies to therapeutically leverage this phosphatase to suppress tumor growth.

## Graphical Abstract

**Graphical Abstract:**
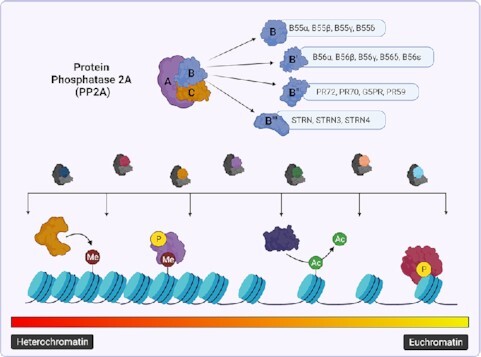
The diverse family of PP2A holoenzymes dynamically regulates the cancer epigenome.

## INTRODUCTION

Aberrant epigenetic regulation of the cancer genome contributes to a wide range of phenotypes, including therapeutic resistance and cellular plasticity (1). In normal cells, control of gene expression through epigenetic regulatory mechanisms allows for rapid cellular responses to various microenvironmental signals. However, oncogenic mutations can significantly alter the epigenetic state of cells, leading to extensive chromatin remodeling, aberrant activation of oncogenes or repression of tumor suppressor genes. These alterations can further contribute to therapeutic resistance and increased survival under unfavorable conditions (2). Further, epigenetic plasticity (either restrictive or permissive) can significantly influence tumorigenic phenotypes, including cell state (e.g. epithelial-to-mesenchymal transition [EMT]) and intratumoral heterogeneity (2,3). Given that epigenetic dysregulation frequently occurs in tumors and significantly contributes to oncogenesis, the therapeutic inhibition of epigenetic pathways that control genome accessibility has shown great clinical promise (4). However, many questions remain regarding the mechanisms that contribute to cancer cell epigenetic states.

In addition to specific DNA sequences that influence gene transcription, there exists a higher order structure to the genome that provides an additional layer of regulation to maintain cellular homeostasis. The nucleosome comprises DNA wrapped around a histone octamer (two copies of H2A, H2B, H3 and H4), which shields the associated DNA from the transcriptional machinery. Reversible epigenetic marks, such as DNA methylation or posttranslational modifications (PTMs) on histones, allow for dynamic changes in the genome without altering the DNA sequence. In response to repressive epigenetic marks, histones will condense to form heterochromatin, or ‘silent’ chromatin regions. Active epigenetic marks help direct remodeling complexes to specific sites of DNA to displace histones and expose critical coding regions, providing additional security that only genes in euchromatic, ‘active’ chromatin regions are readily expressed. Densely packed regions of chromatin are moved to the nuclear lamina to make room for the transcriptional machinery within areas of open chromatin. This dynamic spatial organization, or 3D architecture, generates topologically associated domains (TADs) within the nucleus. During tumorigenesis, the epigenetic regulation of these various chromatin states is frequently altered, significantly contributing to cancer phenotypes and therapeutic response (5–7). However, the mechanisms that regulate this balance are highly complex and context dependent, making the identification of specific epigenetic alterations challenging.

There is a diverse array of cellular complexes that balance the deposition, reading, and removal of epigenetic marks to rapidly alter gene expression based on changes in the cellular environment. These epigenetic modifiers can be divided into three main categories: ‘readers’, ‘writers’ and ‘erasers’. Epigenetic ‘writers’ and ‘erasers’ deposit or remove epigenetic marks, such as methylation, on DNA and histones (5,8). Epigenetic ‘readers’ interpret deposited marks and either recruit transcriptional co-factors or additional chromatin remodeling complexes to specific sites of DNA (5). Together, these modifiers function in a delicate balance to maintain normal gene expression. Epigenetic dysregulation disrupts this balance, contributing to the abnormal activation of oncogenic signaling networks and initiation of tumorigenesis (9). There is a growing body of research indicating that the epigenetic state of cancer cells is significantly impacted by protein phosphorylation (10–13). Kinases and phosphatases rapidly and reversibly modify proteins in response to cellular stimuli, ultimately altering protein activity, localization and binding partners. Critical phosphorylation sites have been identified on epigenetic modifiers and histones that impact their function and/or cellular localization. However, the specific contribution of these phosphorylation sites is still poorly understood.

Kinase inhibitors have been a primary focus for anti-tumor therapeutics contributing to the rise in personalized medicine (14). However, phosphatases play a significant and underappreciated role in suppressing oncogenic signaling and are emerging as novel targets for therapeutic compounds (15,16). Protein Phosphatase 2A (PP2A) is a serine/threonine phosphatase that accounts for approximately 50% of global phosphatase activity (17). The PP2A holoenzyme is composed of three subunits: the scaffolding (A), regulatory (B) and catalytic (C) subunits. While the A and C subunits only contain two different isoforms, the B subunit contributes to a majority of PP2A’s functional diversity with 16 characterized B subunits, many of which contain additional splice variants (15). This array of subunits enables the formation of over 90 distinct and potentially functionally diverse holoenzymes (18). As a whole, the PP2A holoenzyme is categorized as a tumor suppressor due to its negative regulation of many common oncogenes including c-MYC, BCL2, ERK and AKT (19–22). Consistent with this role, PP2A activity is often suppressed in cancer (23,24). Of all the PP2A subunits, PPP2R1A, the PP2A Aα scaffolding subunit, has the highest mutation rate occurring in ∼1% of all cancers (25). These mutations suppress global PP2A activity by disrupting B or C subunit binding and have been shown to drive transformation and tumor growth (26,27). Similarly, the epigenetic silencing of the PP2A B subunit, B55β (PPP2R2B), through DNA hypermethylation results in similar phenotypes, further supporting an important role for PP2A in oncogenesis (28,29). While genetic/epigenetic loss of PP2A function does occur and represents an interesting biomarker for aggressive disease, it is relatively rare and not the primary mechanism by which cancer cells inhibit PP2A activity (24). Instead, tumor cells negatively regulate the PP2A holoenzyme through aberrant expression of endogenous inhibitors and PTMs, which alter PP2A activity, composition, and subcellular localization (30). As a variety of epigenetic regulators have been identified as PP2A targets (Table 1), the dysregulation of PP2A can significantly impact gene transcription, genomic instability and post-translational signaling during oncogenesis. In light of these findings, the therapeutic re-activation of PP2A has emerged as a novel anti-tumor strategy to mitigate oncogenic signaling on multiple levels. Several compounds have been identified to have indirect PP2A activating properties (e.g. FTY720), and small molecules that directly target the PP2A holoenzyme (e.g. SMAPs and iHAPs) are quickly taking the spotlight. However, recent studies have identified pro-tumorigenic roles for specific PP2A subunits, indicating that PP2A inhibitors (e.g. LB100) may also have a place in the clinic (Table 2).

**Table 1.** Epigenetic targets of PP2A.

PP2A Target	Associated B-Subunit	S/T Residue	Opposing Kinase(s)	Effect of Dephosphorylation	References
PRR14	B56a contains SLIM motif and may interact with other B56 family members	S242, T266, T270, S277	Not determined	Promotion of proper localization of heterochromatin to nuclear periphery	(39)
H3	Not determined	S10	AURKA, AURKB, IKKα, JNK, AKT1, MAP3K8, MSK1/2, PIM1	Decreased output of MYC and MYC-related gene targets; Decrease in cell proliferation	(54,57,185)
Lamin A/C	Possibly B56 family members as phosphorylation status changes in response to CIP2A expression	S22, S628	CDK1/CyclinB	Head-to-tail polymerization of Lamin A/C, proper Lamin distribution at nuclear envelope	(41–43)
BRD4	Not determined	S484/S488	CK2	Nuclear localization; Negative regulation of BRD4-associated gene transcription	(68,69)
SWI/SNF	Possibly B55α, only identified in *C. elegans*	Not determined	ERK1	Activation of SWI/SNF complex activity, Mitotic Exit	(186,187)
HDAC2	Not determined	S394	CK2α1	Negative regulation of hypertrophic response in cardiomyocytes	(188)
HDAC4	B55α	S298, S246, S467, S632	PKCϵ, SIK1, SIK2, CaMKII, PKD	Negative regulation of interaction with 14-3-3; Nuclear import of HDAC4; Fibroblast differentiation; Increased glucose uptake; amelioration of neuropathic allodynia; promotes neuronal apoptosis	(76,95,101,189–194)
	B56	Not determined	Not determined	Ensures proper chromosomal segregation during mitosis in p53-null cells	(77)
HDAC5	B55α	S259, S279, S498	SIK1, CaMKII, PKD, PRK1	Nuclear localization; Negative regulation of interaction with 14-3-3	(78,81,189,193–196)
HDAC7	B55α	S155, S181, S321, S449	PRK1	Negative regulation of interaction with 14-3-3; Proper endothelial vessel formation	(75,80,196)
PRMT1	Not determined	S297	Not determined	Inhibition of PRMT1 activity; Decreased methylation of H4 (H4R3me2); HBV-mediated inhibition of INF-α response	(113–115,197)
PRMT5	Not determined	S355	Not determined	Inactivation of PRMT5 methyltransferase activity	(120)
H2A.X	B56ϵ	S129	ATM	Antagonizes DNA damage repair initiation; promotes DNA damage resolution	(115,116,198,199)
TET2	B55α	S99	AMPK	Antagonize stability of TET2	(128)
RNA Pol II	Integrator INTS8, INTS6	S2, S5, S7	CDK9	Negative regulation of transcriptional initiation and elongation	(131,132)
SPT5	Integrator INTS8	S666	CDK7, CDK9	Impaired ‘pause, release’ mechanism of RNA Pol II	(141)

**Table 2.** Therapeutic modulators of PP2A activity.

PP2A modulators	Target	Phosphatase activity	Ongoing Clinical Trials	Application	References
Ceramide	SET	Activating	NCT04716452: Phase I	Acute Myeloid Leukemia	(150,151,200)
FTY720 (Fingolimoid)	SET	Activating	NCT03941743: Phase I	Breast Cancer	(201,202)
			FDA Approved	Multiple Myeloma	
			FDA Approved	Mantle Cell Lymphoma	
			NCT05137860: Phase IV	Acute Lymphoblastic Leukemia	
Bortezomib	CIP2A	Activating	NCT01371981: Phase III	Acute Myeloid Leukemia	(203–205)
			NCT03509246: Phase II	Ovarian Cancer	
			NCT01142401: Phase II	Breast Cancer	
			NCT00479128: Phase I	Urothelial Cancer	
LB100	PP2Ac	Inhibiting	NCT03027388: Phase II	Glioblastoma	(174,206)
			NCT04560972: Phase I	Small Cell Lung Cancer	
OP499	SET	Activating	Pre-clinical		(50,201,207)
iHAP1	PP2A-B56ϵ	Activating	Pre-clinical		(169)
DT061	PP2A-B56α, PP2A-B55α	Activating	Pre-clinical		(169–171,173,208,209)

The function of PP2A and its contribution to tumorigenesis is highly dependent on the composition of the PP2A holoenzyme. Here, we review the complex mechanisms by which PP2A regulates the epigenetic state of cancer cells and potential ways to leverage this activity for anti-cancer therapeutics.

## REGULATION OF HISTONE AND CHROMATIN DYNAMICS BY PP2A

The loss of normal chromatin regulation leads to irregular nuclear morphologies, heterochromatin redistribution and deregulated transcriptional programs, emphasizing the importance of understanding how chromatin states are dynamically regulated (31). Chromatin remodeling requires a coordinated effort between epigenetic regulators and transcriptional complexes and is largely regulated through epigenetic marks within the c-terminal tails of core histones. These marks include acetylation, methylation, ubiquitination and phosphorylation; However, the full impact of protein phosphorylation on the dynamics of chromatin architecture and chromatin reorganization is understudied and still being explored in the field (13).

### Chromatin architecture

Heterochromatic regions are formed in response to repressive histone modifications, such as histone H3 lysine 9 di- and trimethylation (H3K9me2/me3) (32,33). These marks promote the sequestration and anchoring of heterochromatin to the nuclear lamina and is driven through the interaction between chromatin and intermediate filament proteins or nuclear Lamins (Lamin A, Lamin C, Lamin B1 and Lamin B2) (34). Importantly, this higher-order structure plays a critical role in the regulation of gene transcription.

**Figure 1.**
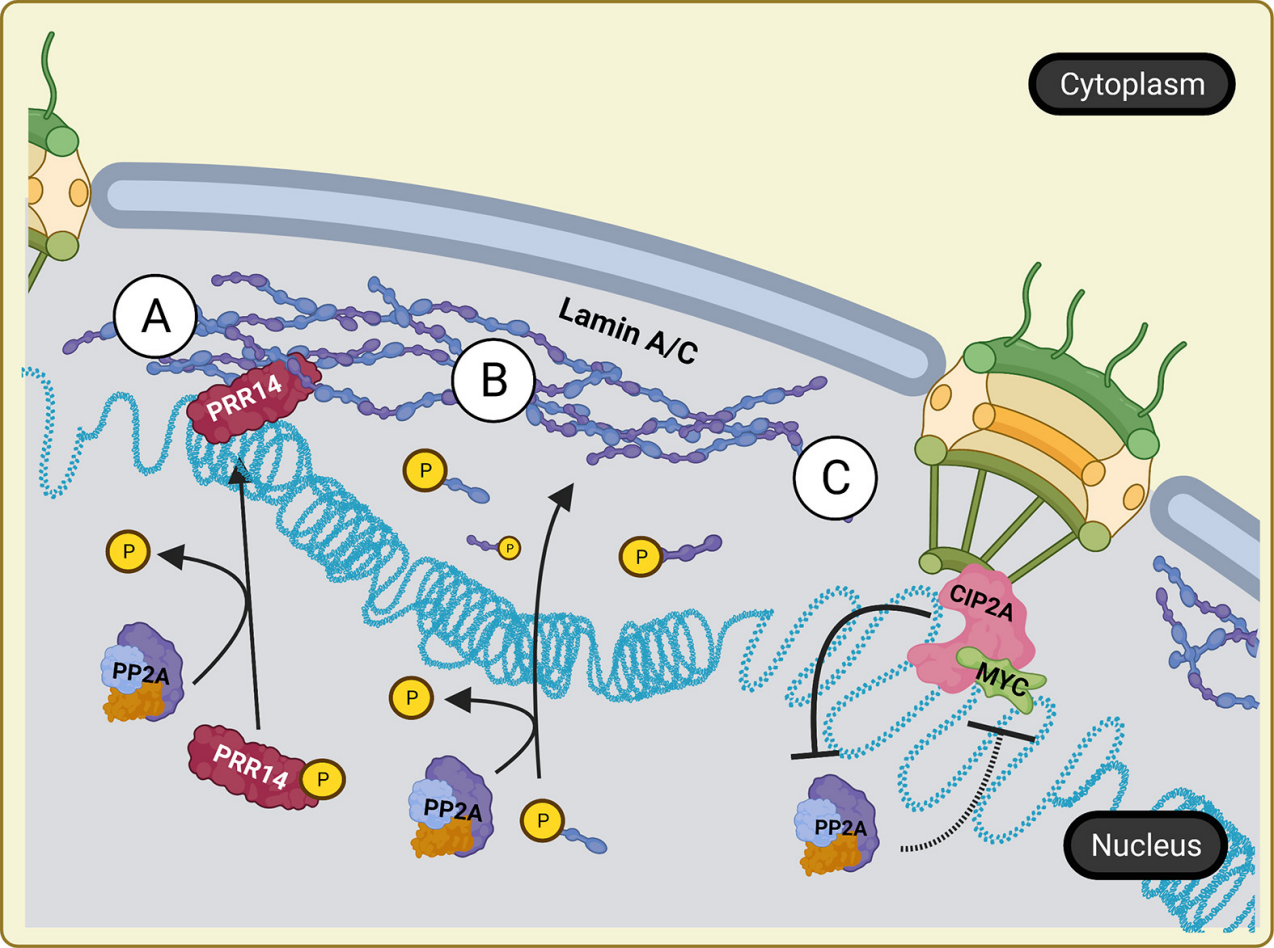
PP2A regulates heterochromatin localization and prevents euchromatic gene expression. (**A**) PP2A dephosphorylates heterochromatin-bound PRR14 to allow HP1–PRR14 interaction and sequestration of heterochromatin to the nuclear lamina. (**B**) PP2A dephosphorylates free nucleoplasmic Lamins to promote Lamin polymerization at the nuclear periphery. (**C**) CIP2A and MYC bind nuclear pore proteins to promote the rapid expression of MYC-dependent genes within associated euchromatin. CIP2A is a known inhibitor of PP2A activity, thereby repressing the ability of PP2A to regulate MYC.

Functionally, nuclear Lamins are critical for maintaining proper nuclear architecture and tethering heterochromatin. Genes located within these Lamin-associated domains at the nuclear periphery exhibit significantly reduced transcriptional levels compared to genes at more central locations (35). Recently, the Proline-Rich Protein 14 (PRR14) was discovered to have a Lamin binding domain (LBD) and associate with heterochromatin protein 1 (HP1) to sequester heterochromatin to the nuclear lamina during interphase and mitotic exit (36). Genetic knockdown of PRR14 results in abnormal nuclear morphologies similar to those seen in cancer cells, suggesting that the localization of heterochromatin to the nuclear lamina through PRR14–HP1 interaction is critical for proper chromatin organization. Dunlevy *et al.* demonstrated that PRR14 contains a conserved short linear motif (SLiM) consistent with PP2A-B56 substrates. This SLiM motif drives the association of PRR14 and PP2A complexes containing B56α (PP2A-B56α) (37,38). PRR14 interaction with PP2A-B56α results in the dephosphorylation of the PRR14 LBD, allowing PRR14–HP1 interaction and localization of heterochromatic regions to the nuclear lamina (Figure 1A) (37,39). Ablation of the PP2A SLiM resulted in a redistribution of PRR14 to the nucleoplasm, suggesting that PP2A is a key regulator of heterochromatin spatial organization within the nucleoplasm.

Additionally, direct phosphorylation of Lamin proteins during mitosis promotes depolymerization and breakdown of the nuclear lamina so that cells may properly undergo cell division (40). Both PP2A and protein phosphatase 1 (PP1) are required for the dephosphorylation of Lamin proteins and the subsequent reassembly of the nuclear envelope (40,41). Phosphorylation of Lamin A/C at serine 22 (pS22) by protein kinases, such as Protein Kinase C and Cyclin Dependent Kinase 1, results in Lamin depolymerization and nucleoplasmic localization (40). This dynamic turnover has been implicated in several cancer processes independent of mitosis (40). Interestingly, nucleoplasmic Lamin has been shown to localize to specific promoters to increase gene transcription (42). This unique cellular function is regulated by the Lamin phosphorylation state, suggesting that there exists a more complex role for Lamins in cancer where phosphatases and kinases are dysregulated. Knockdown of the PP2A A subunit in HeLa cells results in a ten-fold increase in two major phosphorylation sites of Lamin A/C, pS22 and pS628 (41), resulting in nucleoplasmic localization (43). Correct Lamin distribution is important for proper sequestration and silencing of heterochromatic regions; therefore, these results indicate that PP2A suppression may lead to a spatial redistribution of chromatin and expanded gene transcription (Figure 1B). Cancer cells often exhibit atypical nuclear morphologies including increased nuclear to cytoplasmic ratio and irregular nuclear shape as a result of abnormal distribution of lamina and chromatin (44). Given the importance of phosphorylation in the breakdown and reassembly of nuclear lamina, phosphatase deregulation may significantly contribute to these abnormal nuclear morphologies.

### Chromatin organization and transcriptional regulation

In addition to anchoring heterochromatic regions, Lamins also contribute to the distribution of nuclear pore complexes (NPCs) across the membrane and the transcriptional regulation of genes associated with these structures. Although the majority of DNA associated with the nuclear lamina is heterochromatic, the regions surrounding the NPC are commonly euchromatic and highly transcribed (45). NPC-associated genes have been implicated as immediate-early genes (IEGs) which are rapidly transcribed in response to cellular stimulus and are often oncogenic in nature (46). The proto-oncogene c-MYC (MYC) is a transcription factor and an IEG that regulates a wide range of genes. The active, phosphorylated form of MYC has been shown to associate with several NPC proteins, including nucleoporins TPR and Nup153 (47,48). The localization of MYC to this region is associated with a more accessible chromatin state and increased expression of genes involved in migration, proliferation and survival (48). Aberrant MYC expression and/or activation can drive tumorigenesis in most tissue types, highlighting the importance of MYC regulatory pathways. Therefore, to prevent MYC from becoming oncogenic, its activity and expression are highly regulated at the transcriptional, translational and post-translational levels (49). PP2A-B56α negatively regulates a critical MYC phosphorylation site serine 62, which is responsible for MYC’s post-translational stability and activity (20,48,50). Further complicating the role of phosphatases in MYC function, PP2A-B55α positively regulates MYC protein activity and stability through threonine 58 (51). As PP2A likely localizes to the nuclear lamina, PP2A may be an essential regulator of MYC activity at the NPC (Figure 1C) (47,48). Consistent with this hypothesis, the PP2A inhibitor, Cancerous Inhibitor of PP2A (CIP2A), has demonstrated co-localization with MYC and Lamin A/C at the NPC (47). However, CIP2A has been identified to only interact with PP2A complexes that contain B56 family subunits (52). Therefore, only PP2A-B56α, not PP2A-B55α, can be sequestered through CIP2A binding. The presence of CIP2A and MYC at the NPC signifies that the tumor suppressive activity of PP2A-B56α is likely inhibited, potentially promoting oncogenic signaling though a PP2A-B55α-MYC axis. Moreover, knockdown of CIP2A preferentially reduces phosphorylated, active MYC at the NPC but has no effect on MYC phosphorylation within the nucleoplasm (47). These studies set up a dynamic interplay between PP2A and MYC, where PP2A-B56 inhibition allows for the rapid transcription of MYC target genes at the NPC. This phenomenon implicates PP2A in regulating the spatial organization of subnuclear transcriptional domains.

Together, these studies support a model where CIP2A-mediated inhibition of PP2A at the nuclear periphery to facilitate the oncogenic activities of MYC. However, the PP2A subunits that are involved in the process and the contribution of Lamin A/C phosphorylation to association with oncogenic phenotypes need to be further explored.

### Histone-mediated transcription

Phosphorylation of histone H3 (H3) at the serine 10 residue (pH3S10) provides a docking site for chromatin modifiers and increases the acetylation of neighboring residues (53). This PTM is commonly found at sites of active transcription and contributes to the expression of IEGs including c-FOS, c-JUN and MYC, indicating that phosphorylation at this site may contribute to tumorigenesis (54–56). Phosphorylation of the H3S10 residue is elevated in RAS-transformed cells and is associated with poor outcome in multiple cancers (57). Several kinases are known to contribute to the phosphorylation of H3S10, including Aurora kinases, Mitogen and Stress activated protein Kinase 1/2 (MSK1/2), and Proviral Integration site for Moloney murine leukemia virus (PIM-1) kinase (Table 1) (57,58). In particular, PIM-1 has been implicated in cancer proliferation, therapeutic resistance, and contributes to tumorigenesis in multiple tissues (59). Phosphorylation of H3S10 by PIM-1 significantly increases the transcriptional activation of MYC, upregulating approximately 20% of MYC transcriptional targets and leading to oncogenic transformation (58,60,61). Given the importance of pH3S10 to oncogenic transcription and transformation, therapeutic methods to reduce this modification could have significant clinical benefit. Both PP1 and PP2A dephosphorylate the H3S10 site, providing an essential balance to this PTM (54,60). Consistent with these findings, inhibition of PP2A/PP1 by Okadaic Acid (OA) or knockdown of A and C components of the PP2A holoenzyme promote chemical carcinogenesis by preventing PP2A from dephosphorylating the H3S10 residue (54). In addition to H3S10 dephosphorylation, the PP2A-B56β holoenzyme also negatively regulates the stability of PIM-1 (60). Knockdown of B56β leads to a 4-fold increase in PIM-1 half-life, implicating PP2A as a direct and indirect regulator of the H3S10 phosphorylation site (60).

## INTERPLAY OF PP2A AND CHROMATIN REMODELING COMPLEXES

PP2A is reported to alter the activity of chromatin remodeling complexes that regulate or ‘read’ histone epigenetic marks in addition to the direct dephosphorylation of histones. Aberrant regulation of PP2A holoenzyme composition and activity alters the precise signaling that methylation- and acetylation-associated complexes provide, further contributing to oncogenesis by indirectly influencing euchromatic region accessibility.

### PP2A regulation of acetylation

Histone acetylation primarily results in the relaxation of the chromatin and increased transcription of nearby genes. Acetylation marks are added and removed by histone acetyltransferases (HATs) and histone deacetylases (HDACs), respectively, which function in a coordinated effort to maintain normal transcriptional programs. While increased acetylation is often associated with cancer, elevated levels of HDAC activity can similarly contribute to tumor phenotypes by silencing key tumor suppressor genes, such as p53 (5,62,63). Despite the critical role of acetylases in regulating gene transcription, therapeutic inhibition of acetylases has shown limited clinical success as single agents (64). This lack of efficacy is likely due to heterogeneous expression (65) and function of chromatin remodelers (66). The cellular localization and activity of acetylation complexes are controlled through phosphorylation, adding an additional layer of regulation to global transcriptional programs and a potential mechanism by which cells can become resistant to HAT/HDAC inhibition (67). Therefore, the use of HAT/HDAC inhibitors may benefit from combinatorial treatment with specific phosphatases that aid in the regulation of acetylases.

### ‘Readers’ of acetylation

PP2A also regulates the activity of lysine acetylation ‘readers’, including the Bromodomain and Extraterminal (BET) protein, Bromodomain-containing 4 (BRD4). BRD4 accumulates at euchromatic regions to promote gene transcription through the recruitment of transcription factors to the transcription start site or as a part of super-enhancer complexes (65). Phosphorylation of BRD4 by casein kinase II (CK2) is reported to increase the association of BRD4 with activating acetylation marks, making hyperphosphorylation of BRD4 a correlate of poor prognosis in multiple cancers (68–70).

Given the prominent role that BET proteins play in gene transcription, a considerable amount of effort has gone into the development of BET inhibitors with varying success. In 2011, Delmore *et al.* unexpectedly demonstrated that rather than global transcriptional repression, the BET inhibitor, JQ1, only modified the expression of a limited number of genes, indicating that specific genes displayed unique susceptibility to BET inhibition. Of the affected targets, the MYC super-enhancer was acutely reliant on BRD4-dependent transcriptional activation (71). MYC contains large intrinsically disordered regions and lacks tangible binding pockets, making the direct therapeutic targeting of this potent transcription factor challenging (72). Thus, BET inhibitors opened a door to new MYC therapeutic strategies (71).

**Figure 2.**
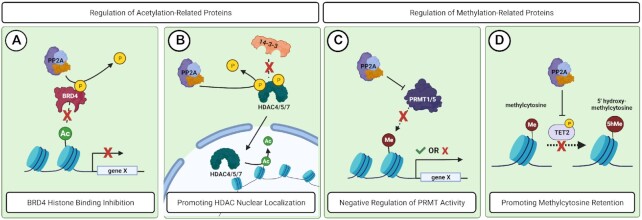
PP2A regulates proteins that contribute to acetylation and methylation of epigenetic targets. (**A**) PP2A dephosphorlates BRD4, preventing BRD4 from binding to acetylated residues and promoting gene transcription. (**B**) PP2A dephosphorylates HDAC 4/5/7 and prevents 14-3-3 binding, allowing HDAC nuclear localization and subsequent deacetylation of histones. (**C**) High PP2A activity is correlated with low PRMT1/5 methylase activity at the H4R3me2 methylation site. The impact of this regulation on gene transcription is cancer dependent. (**D**) PP2A dephosphorylates TET2 and reduces TET2 stability. This action antagonizes efficient methylcytosine removal.

While BET inhibitors have shown efficacy across various cancers, development of resistance was an immediate concern. As phosphorylation of BRD4 is counteracted by PP2A (Figure 2A), suppression of PP2A can increase BRD4 activation and resistance to BET inhibitors, including JQ1 (41,73). Therefore, restoring PP2A activity may be important for maximizing the therapeutic efficacy of BET inhibitors. For example, the combination of the PP2A-activating compound, perphenazine and JQ1 resulted in a synergistic loss of triple negative breast cancer cell viability (69,73). In addition to the transcriptional control of MYC, PP2A also directly post-translationally regulates MYC protein stability (20). In cancer, PP2A activity is commonly suppressed, leading to increased MYC phosphorylation and transcriptional activity (74). Together, these studies place PP2A as a critical mediator of both epigenetic and post-translational regulation of MYC during tumor progression.

### ‘Erasers’ of acetylation

Histone deacetylase (HDAC) complexes play an important role in suppressing gene transcription by removing acetylation marks and promoting formation of heterochromatin. Given that deregulation of HDAC activity can contribute to either tumor suppressive or oncogenic signaling, proper HDAC nuclear localization is extremely important for cellular homeostasis (11). Class IIa HDACs: HDAC4, HDAC5 and HDAC7 have all been reported to be substrates of PP2A (75–78). Phosphorylation of these complexes creates binding sites for the 14-3-3 chaperone proteins, sequestering HDAC 4/5/7 to the cytosol (78–81). Dephosphorylation of these sites by PP2A promotes the rapid nuclear localization of HDACs and the suppression of respective target genes (Figure 2B).

PP2A-mediated regulation of HDAC complexes greatly depends on the composition of the PP2A holoenzyme (24). In contrast to the known tumor suppressive role of the B56α subunit, a growing number of studies indicate that the B55α subunit may play a pro-oncogenic role (51,82–86). Consistent with these findings, the PP2A-B55α holoenzyme has been shown to regulate angiogenesis by increasing yes-associated protein 1 (YAP1) transcription in response to vascular endothelial growth factor receptor (VEGFR) signaling and drive the accumulation of hypoxia inducible factor 1 subunit alpha (HIF1a) (84,87). Additionally, PP2A-B55α has been implicated in promoting endothelial vascular integrity and proper lumen architecture by positively regulating HDAC7 nuclear import and promoting HDAC7-dependent transcriptional programs (75,80). Although these results implicate B55α in the positive regulation of angiogenesis, treatment with the small molecule inhibitor of PP2A, LB100, resulted in increased angiogenesis and vascular permeability in hepatocellular carcinoma and pancreatic cancer (88,89). As LB100 is a ubiquitous catalytic inhibitor of PP2A, this discrepancy highlights the importance of understanding the individual contribution of PP2A B subunits to tumor phenotypes.

HDAC4 signaling contributes to several cancer phenotypes, including increased proliferation and suppression of differentiation transcriptional programs (90). Although HDAC4 nuclear localization by PP2A has been shown in other cell types, the contribution of this interaction to tumorigenesis is not well characterized. HDAC4 promotes proper chromosomal segregation during mitosis in p53-null cancer cells (77). This function is in part dependent on the interaction between HDAC4 and PP2A-B56α, a key regulator of the metaphase-to-anaphase checkpoint of mitosis (91–94). Consistent with PP2A’s regulation of HDAC function, loss of PP2A-B56α expression resulted in chromosomal segregation defects and phenocopied HDAC4 knockdown. However, the direct impact of altered PP2A-B56α function on HDAC4 localization and transcriptional programs is unknown. It is plausible that decreased PP2A activity combined with genetic loss of p53 may lead to the propagation of cells with a high degree of genomic instability, ultimately promoting tumorigenesis, but further studies are needed.

Regulation of HDAC4 by PP2A has also been shown to impact the differentiation state of fibroblasts (95). Reprogramming of fibroblasts by tumor cells has emerged as a critical component of tumor biology, with infiltration and activation of fibroblasts contributing to proliferation, metastasis and therapeutic resistance in several cancer types (96–99). In response to extracellular signals such as transforming growth factor beta (TGFβ), fibroblasts can take on a myofibroblastic phenotype, displaying increased expression of alpha smooth muscle actin (αSMA) and extracellular matrix proteins (100). This reprogramming is dependent on the nuclear localization of HDAC4, as knockdown of HDAC4 prevents TGFβ−induced αSMA expression and the suppression of PP2A rescues this phenotype (95,100,101). PP2A activating compounds have been shown to reduce oncogenic signaling in cancer cells; however, this phenomenon may not be true in fibroblasts. PP2A activation in cancer associated fibroblasts (CAFs) may further promote oncogenic activities of HDAC regulation given that activation of CAFs can positively contribute to fibrosis. This event can further complicate therapeutic treatment and stresses the importance of understanding PP2A holoenzyme- and tissue-specific function.

### PP2A regulation of methylation

Histone methylation marks are extremely diverse in function and can be associated with promotion or inhibition of gene transcription. The role of each methylation mark depends on the genomic site, the presence of other neighboring histone PTMs or epigenetic marks, as well as the number of methyl groups deposited on any given residue (mono-, di-, tri-methylation) (8). Similar to phosphorylation and acetylation, the methylation of various histone residues is regulated by methyltransferases and demethylases that work in tandem to maintain homeostatic gene expression. Importantly, mutation or dysregulation of these complexes can lead to transformation and tumorigenesis (102,103).

### ‘Writers’ of histone methylation

While methylation of lysine residues (K) is more common, the field of arginine (R) methyltransferases has significantly expanded within the past decade. There are three subclasses of protein arginine methyltransferases (PRMTs): (i) asymmetric dimethylarginine (ADMA), (ii) symmetric dimethylarginine (SDMA) and (iii) monomethyl arginine (MMA) (103). All PRMTs deposit monomethyl marks, but symmetrical or asymmetrical dimethylation is dependent on the specific PRMT class, adding another layer of complexity to the signaling (104). PRMTs dynamically contribute to the co-activation or co-repression of genes by promoting the recruitment of transcription factors or other epigenetic complexes (105).

PRMT1 is the predominant ADMA methyltransferase, accounting for approximately 85% of total arginine methyltransferase activity (106). PRMT1 primarily asymmetrically dimethylates the H3R4 residue (H3R4me2a), which is associated with transcriptional activation (107,108). High levels of H3R4 methylation have been positively correlated within tumor grade and risk of recurrence in prostate cancer (109). Additionally, PRMT1 has oncogenic roles in colorectal cancer, breast cancer and pancreatic cancer (110–112). PP2A has been reported to negatively regulate PRMT1 through dephosphorylation at S297, supporting a possible tumor suppressive role for PP2A in the context of PRMT1 regulation (113). However, in Hepatitis C (HepC)-induced hepatocellular carcinoma (HCC), PP2A activity is upregulated in the presence of the HepC virus, aiding in viral replication and paradoxically promoting tumorigenesis of HepC-HCC (114). The combination of high PP2A activity and low PRMT1 activity in HepC-HCC has also been implicated in promoting anchorage independent growth and clonogenic proliferation through dysregulation of histone H4R3 methylation (Figure 2C) (115,116). Further, a follow-up study determined that the inhibition of PP2A with LB100 enhanced the effect of chemotherapies in HepC-HCC, potentially pointing to a unique role for PP2A in this tumor type (115). Although the specific PP2A B subunits responsible for these phenotypes were not explored, these results implicate PP2A in a novel pro-tumorigenic role in HepC-HCC.

PRMT5 is the predominant SDMA methyltransferase and is generally associated with gene repression (103). There is an abundance of evidence suggesting that PRMT5 plays an oncogenic role in many cancers such as prostate cancer, pancreatic cancer, and colorectal cancer (117–119). In adult T-cell leukemia (ATL), PP2A has been reported to negatively regulate PRMT5 through the dephosphorylation of S355. This interaction is mediated by N-MYC downstream regulated gene (NDRG2), which acts as an adaptor between PRMT5 and PP2A (120,121). When NDRG2 expression is low, PRMT5 is reported to promote cell growth and prevent apoptosis (120). Therefore, PP2A-activating compounds may be effective in cancers with functional NDRG2 and may synergize with PRMT5 inhibiting compounds, while NDRG2 deletions may render this strategy ineffective. Conversely, in glioblastoma, PRMT5 exhibits a tumor suppressive role, while NDRG2 is reported to have oncogenic properties (122,123). Consistent with the findings, treatment of glioblastoma with PP2A-inhibitor LB100 has an anti-tumor effect in the context of PRMT5 regulation (122). Therefore, while the mechanism by which PP2A regulates PRMT5 is the similar, the biological outcome of this signaling is contradictory between these two cancers and supports the use of alternative therapeutic approaches.

### ‘Erasers’ of DNA methylation

In addition to histones, cytosine nucleotides can also be directly methylated. Methylated cytosine results in the eventual formation of large stretches of CpG islands, or areas of dense DNA methylation, normally found at promoters of actively repressed genes. Reversal of methylated cytosine is a process that requires multiple oxidative reactions by ten-eleven translocation (TET) proteins and conversion to an abasic site that can be recognized for base excision repair (124). The TET proteins predominantly function as tumor suppressors and are important for sequential oxidation of 5’-methylcytosine for restoration of the unmodified cytosine base (125). The stability of TET proteins is regulated by phosphorylation (126). AMP activated kinase (AMPK) is reported to phosphorylate TET2 at serine 99 (S99), driving the interaction with 14-3-3 and increasing global DNA 5-hydroxymethylcytosine levels. PP2A dephosphorylates TET2 at S99, antagonizing TET2 stability and activity (Figure 2D) (127,128). The PP2A-TET2 interaction is mediated specifically through the PP2A-B55α complex, consistent with an oncogenic role for this PP2A B subunit (128). The importance of this phospho-regulation is underscored by the fact that mutations in TET2 often occur in the S99 region (129), causing aberrant formation of CpG islands and silencing of genes that are important for homeostatic regulation.

### Direct transcriptional regulation

#### PP2A-integrator complex (INTAC)

**Figure 3.**
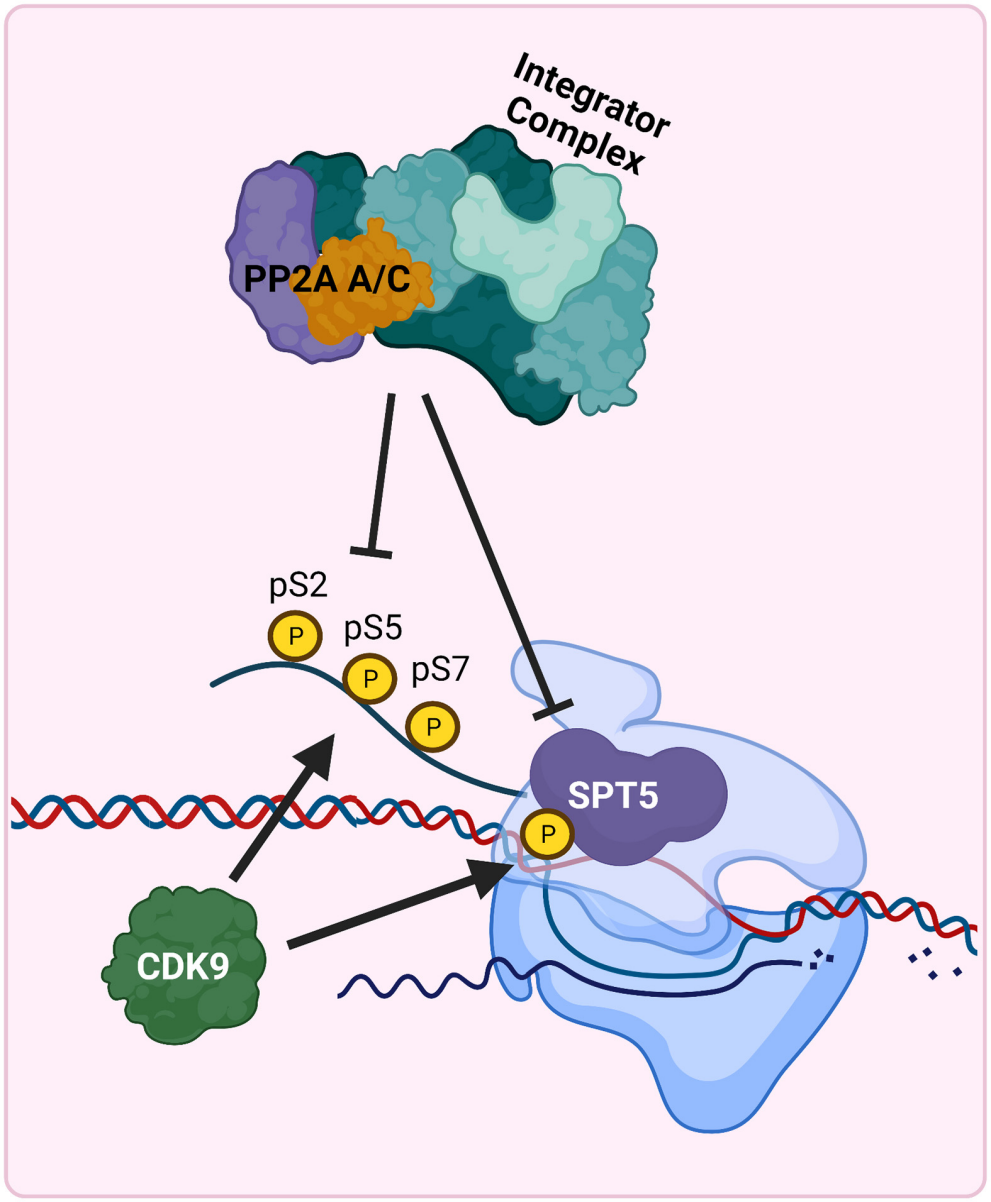
PP2A contributes to regulation of transcription as part of the Integrator-PP2A (INTAC) complex. The PP2A A/C subunits bind to the Integrator complex and promote dephosphorylation of S2/5/7 residues on the tail of RNA Polymerase II in addition to SPT5. These actions prevent efficient transcriptional initiation and elongation of RNA transcripts. INTAC dephosphorylation of RNA Polymerase II and SPT5 is counteracted by CDK9 phosphorylation.

The canonical PP2A heterotrimeric complex consists of an A/C dimer paired with a regulatory B subunit, which dictates substrate specificity. Recently, multiple papers have been published describing a non-canonical PP2A holoenzyme called INTAC in which the 14-subunit Integrator complex (130) displaces canonical B subunits and directly associates with the PP2A A/C heterodimer (131–133). Consistent with the characterized role of the Integrator complex (130,134), INTAC localizes to RNA polymerase II (RNA Pol II)-bound chromatin and exhibits both endonuclease and phosphatase activity (131). Knockdown of the PP2A C subunit resulted in a genome-wide increase in RNA Pol II phosphorylation at Serine 2, 5 and 7 within the c-terminal tail and increased the expression of genes targeted by INTAC activity (131). Based on the canonical function of these residues, the phosphatase activity of the INTAC complex negatively regulates both transcriptional initiation and elongation (Figure 3) (131). The role of phosphatases and PP2A-integrator function on transcriptional machinery was well summarized by Cossa *et al.* earlier this year (135).

Cancer cells commonly maintain high levels of transcription to accommodate increased proliferation rates and oncogene-induced gene expression (136). This high transcriptional output may represent a cancer-specific vulnerability that can be leveraged through activation of INTAC complex activity. Cyclin Dependent Kinase 9 (CDK9) promotes transcriptional elongation and increases mRNA transcription through RNA Pol II phosphorylation at Serine 2 and 5 (137). Multiple types of solid and hematopoietic malignancies are addicted to elevated transcription rates and are, therefore, sensitive to CDK9 inhibitors (138–140). It was recently discovered that the INTAC complex directly opposes CDK9 phosphorylation and that treatment with a small molecule activator of PP2A (SMAP) synergizes with CDK9 inhibition (132). In addition, SPT5, an essential positive regulator of transcription and a target of CDK9 phosphorylation, has also been observed to be negatively regulated by the INTAC complex, further supporting CDK9 inhibitor/PP2A activator synergy (Figure 3) (133,141). While CDK9 inhibitors alone have demonstrated antitumoral activity in preclinical research, CDK9 inhibitors have been largely unsuccessful in the clinic due to limited drug efficacy and adverse side effects (142). Therefore, combination treatment with CDK9 inhibitors and SMAPs may increase anti-tumor responses while reducing adverse side effects in patients.

## THERAPEUTIC TARGETING OF PP2A IN CANCER

As PP2A regulates a wide range of targets, its activity and holoenzyme composition are tightly controlled through expression, localization, phosphorylation, methylation and association with cellular inhibitors (24). There are three major endogenous cellular inhibitors that regulate PP2A activity: Inhibitor 2 of PP2A (I2PP2A or SET), CIP2A and PME1, all of which have been heavily implicated in tumor promotion and are overexpressed in many cancer types (30). Kauko *et al.* demonstrated that genetic knockdown of SET, CIP2A or PME1 globally increases the sensitivity of cancer cells to a panel of kinase inhibitors, while knockdown of the PP2A A subunit drives global therapeutic resistance (41). These studies also show that CIP2A is predominately cytoplasmic, whereas PME1 and SET are nuclear. Together, these studies implicate PP2A as an important rheostat for therapeutic response and suggest that the spatial regulation of PP2A activity may lead to distinct phenotypes and unique therapeutic combinations depending on cellular context. In an effort to modify PP2A activity in tumors, three main therapeutic strategies have been explored: preventing cellular inhibitors from binding to PP2A, directly nucleating the PP2A holoenzyme or global inhibition of PP2A (Table 2) (143,144).

### Inhibiting the inhibitors of PP2A

SET is a global inhibitor of PP2A that binds to the C subunit of PP2A and inhibits catalytic activity of the PP2A holoenzyme (145,146). High SET expression has been implicated in poor survival outcomes in a diverse set of cancers, including colorectal cancer, breast cancer and hepatocellular carcinoma (147–149). Suppression of SET leads to decreased phosphorylation of proteins involved in histone methylation and chromatin organization, consistent with SET nuclear localization (41). FTY720 (Fingolimod) is a ceramide derivative and sphingosine analog that inhibits the binding of SET to the PP2A holoenzyme, thereby indirectly promoting PP2A phosphatase activity (150,151). FTY720-mediated inhibition of SET has shown promising anti-tumor effects through the activation of PP2A in lung cancer, colorectal cancer and various hematologic malignancies (152–155). In colorectal cancer, FTY720 was found to promote PP2A-mediated degradation of Polycomb Group RING Finger Protein 4, BMI1, thereby preventing Polycomb Repressive Complex 1 (PRC1)-based epigenetic modifications (156). Given that PRC1 is implicated in promoting stem-like states and metastasis in multiple cancers, particularly through BMI1-containing PRC1 complexes, the re-activation of PP2A in this context may help to target relevant cell sub-populations within tumors (157–159). Other studies have indicated that FTY720 can suppress transcription by altering histone modifications, including H3K27 trimethylation, although the direct prevention of PRC1-mediated oncogenesis by PP2A has not been explored (160,161). Despite the anti-tumor effects of FTY720, this compound elicits severe cardiac toxicities among other side effects. An analog compound, SH-BC-893, potentially addresses these issues and is currently in preclinical phases (162,163). While there are ongoing efforts in the cancer field to develop direct small molecule inhibitors of PME1 and CIP2A, these strategies are lagging compared to those that target SET.

### Direct activation of PP2A phosphatase activity

While cellular PP2A inhibitors play a significant role in PP2A function, many have PP2A-independent functions, making the therapeutic inhibition of these factors problematic (164,165). Therefore, a considerable amount of effort has been spent developing direct activators of PP2A. Early in the development of PP2A activators, the phenothiazine class of antipsychotics were found to have antiproliferative effects on cancer cells (166) by indirect activation of PP2A through inhibition of calmodulin (167). Due to off-target effects of D2 dopamine receptor antagonism, phenothiazines could not be used therapeutically in cancer but provided the structural basis for the development of more specific PP2A activators: improved Heterocyclic Activators of PP2A (iHAPs) and Small Molecule Activators of PP2A (SMAPs) (168). These allosteric activators directly increase PP2A activity by nucleating and stabilizing various PP2A holoenzymes (169,170). Interestingly, both iHAPs and SMAPs promote the incorporation of B56 family members (B56ϵ and B56α, respectively) into the complex (169,170). However, studies have suggested that SMAPs, such as DT061 can promote incorporation of other B subunits, including B55α (169). Differences in PP2A complex stabilization have been observed between cancers such as lung adenocarcinoma (170) or in various hematologic malignancies (169). These conflicting results may be due to variances in expression of PP2A complexes or through differences in post-translational modification of PP2A within each unique malignancy (74). SMAPs have shown significant pre-clinical efficacy *in vivo*, both as a single agent and in combination with other compounds but has yet to be assessed in the context of epigenetic-based therapeutics (168).

### Direct inhibition of PP2A phosphatase activity

While most cancers have been shown to be sensitive to PP2A re-activation, there are some contexts in which tumors display increased sensitivity to PP2A inhibition. Lung cancer, pancreatic cancer and leukemias have been reported to be sensitive to therapeutic re-activation of PP2A activity (169,171–173). On the other hand, cancers such as glioblastoma show therapeutic efficacy with PP2A catalytic inhibitors, such as LB100, particularly when used in combination with DNA damaging therapeutics (122,174–177). The combination of PP2A and epigenetic inhibitors are currently being explored and may prove effective in malignancies of the nervous system (122,144). In either context (activation or inhibition), PP2A holds great promise as a therapeutic target as more specific compounds are developed (Table 2). However, the complex regulation of PP2A demands a greater understanding of the context-specific functions of the PP2A holoenzyme for the rational design of new therapeutic compounds and the use of combination therapies.

## DISCUSSION

### PP2A therapeutic targeting challenges

**Figure 4.**
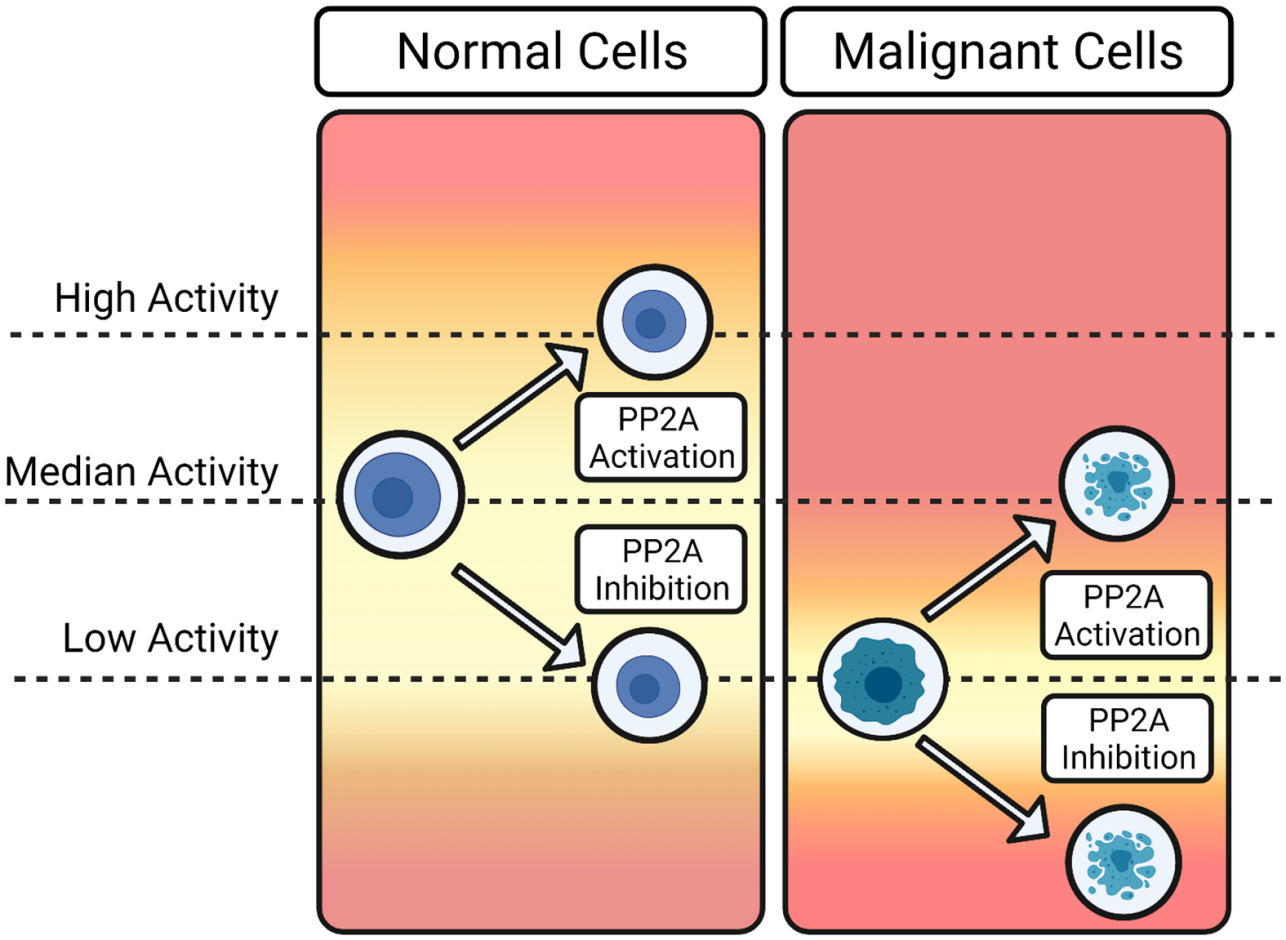
Cancer cells have different thresholds for PP2A activity than normal cells Normal cells (left) maintain median PP2A activity levels and have a larger tolerance for fluctuations in PP2A phosphatase activity. In contrast, malignant cells (right) become addicted to low levels of PP2A activity and, therefore, become more susceptible to fluctuations in activity. Upon therapeutic activation of PP2A, malignant cells cannot withstand PP2A activation returning to baseline levels and the subsequent loss of oncogenic signaling. Similarly, given that malignant cells already function on low levels of PP2A activity, inhibition of PP2A activity results in a loss of survival signals and cell apoptosis. Since PP2A activators and inhibitors display little to no toxicity, this would suggest that normal cells are able to maintain the signals necessary for survival in both contexts.

One of the most exciting aspects of PP2A-centric therapeutics has been the low toxicity observed *in vivo* (170). While the exact mechanism underlying the potential cancer cell selectivity of PP2A targeting agents is still being explored, it is likely that as tumors progress, cancer cells become reliant on low PP2A activity for survival. Therefore, when PP2A is therapeutically activated, cancer cells are unable to compensate for the loss in oncogenic signaling and undergo cell death. In contrast, normal cells, which are predominately quiescent, have a much higher threshold for the amount of PP2A activity that can be tolerated before losing critical survival signals (Figure 4). Intriguingly, HDAC inhibitors seem to function similarly, with cancer cells displaying a unique vulnerability to epigenetic-based therapeutics (178). Ultimately, these findings indicate that cancer cells develop unique regulatory mechanisms that differ from normal cell populations.

Several studies demonstrate that PP2A contributes to the regulation of various epigenetic and transcriptional processes, but most of these studies inappropriately treat PP2A as a singular entity. For example, many studies utilize the phosphatase inhibitor, OA, as proof that PP2A regulates specific signaling pathways. However, OA inhibits both PP1 and PP2A, which confounds experimental conclusions (179). Similarly, very few research studies tease apart the contribution of distinct PP2A holoenzymes from global PP2A activity. As a result of these oversimplifications, several reports indicate potentially contradictory roles for PP2A in cancer epigenetics. Additionally, it is likely that some PP2A subunits can in part compensate for the loss of other subunits. Although variants of B subunits within each of the B subunit families (B55, B56, PR72 and Striatins) have high degrees of structural homology, the targets and function of these B subunits can be distinct. These gaps in knowledge severely limit our understanding of PP2A-dependent phenotypes and complicate the clinical use of PP2A therapeutics.

Just as PP2A is not ‘one’ protein, the localization and function of PP2A complexes may not be restricted to one cellular compartment or be uniform in response. The functional consequence of higher-order chromatin architecture, including TAD domains, phase separation and chromosome territories are all being interrogated in the context of cancer phenotypes (180,181). The presence of these complex nuclear microdomains impacts not only inter- and intratumoral heterogeneity but potentially drives differential regulation of the epigenetic processes by phosphatases in distinct locations. Delineating the spatial and kinetic activities of PP2A will potentially uncover the critical cancer-specific mechanisms verses those that are dispensable.

### Leveraging the PP2A interactome for personalized medicine

Extensive research efforts have been spent determining the consequence of specific phosphorylation sites on protein function. Interestingly, the number of kinase-regulated proteins that have been identified significantly overshadows the number of proteins known to be direct targets of phosphatases, suggesting that the cancer-associated PP2A interactome is far from complete (182). The transient nature of the protein-protein interactions between phosphatases and their substrates has severely limited our ability to identify substrates by traditional methods of detection (e.g. co-immunoprecipitation and mass spectrometry-based assays). To overcome this barrier and make significant strides in PP2A substrate identification, studies are now incorporating more advanced techniques, such as proximity labeling and crosslinking to identify PP2A complexes in live cells. For example, TurboID (a modified version of BioID) has been performed to create an interactome map of specific PP2A B subunits utilizing a unique biotin ligase that covalently label proteins within close proximity (183). This unbiased approach generates a large number of potential targets but lacks spatial information about these interactions and generates a high rate of false positives due to the promiscuous nature of the ligase. Recently, a conditional version of this assay, SplitID, has been developed, in which two proteins each get one half of the biotin ligase and only form a functional ligase when complexed together (184). This strategy has been used to identify targets of specific PP1 complexes but has not yet been used specifically with individual PP2A B subunits.

Proximity ligation assays (PLA) has been used as a targeted approach to identify not only if two proteins interact (phosphatase-substrate) but the location of the complex within the cell. This additional spatial information is invaluable for dissecting potential subcellular domains of phosphatase function (nuclear, perinuclear, cytoplasmic, etc.) and dynamic shifts in protein localization or PP2A holoenzyme composition in response to perturbations such as oncogenic mutation. To decipher transcriptional regulatory complexes, chromatin immunoprecipitation with selective isolation of chromatin-associated proteins (ChIP-SICAP) is now being used to isolate both protein complexes and their associated DNA in the *same* experiment. This unique approach uses crosslinking to stabilize transient protein–protein interactions and could be applied to identify specific PP2A-regulated genes and associated binding partners. Ultimately, the toolbox available to interrogate PP2A interactions is growing rapidly and will no doubt increase our understanding of this complex family of regulators.

As we begin to unravel the context-specific roles of PP2A in the regulation of epigenetic and transcriptional mechanisms, new compounds could be developed to capitalize on the unique functions of individual PP2A B subunits. Already, compounds are emerging that preferentially activate unique PP2A complexes (SMAPs and iHAP1), suggesting that eventually we may be able to tailor therapeutics to specific PP2A-dependent cancer phenotypes (170). Since the dynamic regulation of PP2A holoenzyme composition can greatly differ between tissues and cancer types, this strategy would allow for a more personalized therapeutic approach.
